# Novel applications of motif-directed profiling to identify disease resistance genes in plants

**DOI:** 10.1186/1746-4811-9-37

**Published:** 2013-10-07

**Authors:** Jack H Vossen, Sara Dezhsetan, Danny Esselink, Marjon Arens, Maria J Sanz, Walter Verweij, Estelle Verzaux, C Gerard van der Linden

**Affiliations:** 1 Plant Breeding, Wageningen University and Research Center, Wageningen, Netherlands; 2 Department of Agronomy & Plant Breeding, Faculty of Agricultural Sciences, University of Mohaghegh Ardabili, Ardabil, Iran; 3 Department of Cell Biology and Genetics, University of Alcala, Madrid, Spain; 4 Sainsbury laboratory, Norwich, United Kingdom; 5 Current address: Universidad Técnica del Norte, Ibarra, Equador

**Keywords:** Motif-directed profiling, Resistance gene, Potato, *Solanum berthaultii*

## Abstract

**Background:**

Molecular profiling of gene families is a versatile tool to study diversity between individual genomes in sexual crosses and germplasm. Nucleotide binding site (NBS) profiling, in particular, targets conserved nucleotide binding site-encoding sequences of resistance gene analogs (RGAs), and is widely used to identify molecular markers for disease resistance (*R*) genes.

**Results:**

In this study, we used NBS profiling to identify genome-wide locations of RGA clusters in the genome of potato clone RH. Positions of RGAs in the potato RH and DM genomes that were generated using profiling and genome sequencing, respectively, were compared. Largely overlapping results, but also interesting discrepancies, were found. Due to the clustering of RGAs, several parts of the genome are overexposed while others remain underexposed using NBS profiling. It is shown how the profiling of other gene families, i.e. protein kinases and different protein domain-coding sequences (i.e., TIR), can be used to achieve a better marker distribution. The power of profiling techniques is further illustrated using RGA cluster-directed profiling in a population of *Solanum berthaultii*. Multiple different paralogous RGAs within the *Rpi-ber* cluster could be genetically distinguished. Finally, an adaptation of the profiling protocol was made that allowed the parallel sequencing of profiling fragments using next generation sequencing. The types of RGAs that were tagged in this next-generation profiling approach largely overlapped with classical gel-based profiling. As a potential application of next-generation profiling, we showed how the *R* gene family associated with late blight resistance in the SH*RH population could be identified using a bulked segregant approach.

**Conclusions:**

In this study, we provide a comprehensive overview of previously described and novel profiling primers and their genomic targets in potato through genetic mapping and comparative genomics. Furthermore, it is shown how genome-wide or fine mapping can be pursued by choosing different sets of profiling primers. A protocol for next-generation profiling is provided and will form the basis for novel applications. Using the current overview of genomic targets, a rational choice can be made for profiling primers to be employed.

## Introduction

The majority of the world’s biomass is produced by members of the plant kingdom, and the majority of the other kingdoms are dependent on plants for their survival. This results in a wide range of interactions between species ranging from symbiosis, pathogenicity or herbivory. Plants have evolved multiple mechanisms to cope with these interactions and especially to defend themselves against biotic threats. For their protection against pathogenic microorganisms, plants largely rely on an innate immune system that encodes a wide array of pathogen receptors [1]. Genes encoding receptors that can successfully intercept invasion of a plant by a pathogen are called disease resistance (*R*) genes.

Many *R* genes have now been cloned and characterised from a variety of plants. Based on the molecular structure of the encoded proteins they can be grouped into several classes. These classes include the nucleotide binding leucine-rich repeats proteins (NLR), receptor-like proteins (RLP), receptor-like kinases (RLK), including LRR-kinases and lectin receptor kinases, and intracellular protein kinases (PK) ([2-4]). The NLR class, which is characterised by the presence of two conserved domains, a central nucleotide binding site (NBS) and a C-terminal series of leucine-rich repeats (LRRs), represents the largest class of *R* genes in plants [5]. NLR proteins are located in the cytoplasm, associated with the inner leaflet of the plasmamembrane [6], the endosome [7] or in the nucleus [8]. The NLR class can be divided into two distinct subclasses based on the N-terminal domain structures. The first subclass is characterised by the Toll/interleukin-1/receptor (TIR) domain, which is homologous to the *Drosophila* Toll and mammalian interleukin-1 receptors. The group that does not have a TIR domain, collectively referred to as non-TIR, is very diverse, and often a predicted coiled-coil (CC) structure can be found (reviewed by [4,9]). The class of RLPs, founded by the tomato *Cf-9* resistance genes providing resistance to the fungal pathogen *Cladosporium fulvum*[10], encode plasmamembrane-localised receptors. The extracellular N-terminal domain contains multiple LRRs, while the C terminus contains a single membrane-spanning domain and a short intracellular tail. The RLK class encodes proteins consisting of an extracellular receptor domain connected through a single transmembrane domain to a cytoplasmic serine-threonine kinase domain*.* The RLK class can be divided into different subclasses based on the different extracellular receptor domains, which can contain LRRs, lectin like- or additional domains that can potentially bind pathogen-derived peptides or oligosaccharides. Examples of members of the RLK class are the *Xa21* and *Xa26* genes from rice providing resistance to *Xanthomonas oryzae* pv. *oryzae*[11,12] and the *RFO1* gene from Arabidopsis providing resistance to *Fusarium oxysporum*[13]. The PK class of *R* genes encode only serine–threonine kinases but no transmembrane domains or LRRs. The PK class includes *Pto* from tomato [14] and *PBS1* from Arabidopsis, which provide resistance to different strains of *Pseudomonas syringae*[15]. *Rpg1*, from barley, has two tandem protein kinase domains [16]. In addition, *R* genes are known that cannot be grouped into one of the described classes, like the Rpg5 protein from wheat that contains both NBS and PK domains [17].

For marker-assisted breeding, it is essential to generate molecular markers that are located as close as possible to *R* genes in the genome. In addition, for GM breeding approaches it is essential to generate closely linked markers in order to clone a gene of interest [18]. Many techniques are available to generate such molecular markers. The techniques differ, however, in their efficiency and downstream applications. For *de novo* mapping, it is sometimes preferred to use unbiased marker techniques like DART [19], SSR or AFLP [20], and more recently to use single nucleotide polymorphism (SNP) arrays [21,22]. The presence of several conserved domains in *R* genes provides tools for more biased marker techniques that directly target *R* gene analogs (RGAs). RGAs can be amplified by PCR-based approaches using two (degenerate) primers in conserved domains [23]. Since plant genomes typically contains hundreds of RGAs, PCR-based approaches result in highly complex amplicons requiring additional complexity reduction. Profiling techniques [24] combine a single (degenerate) RGA primer with an adapter ligated to a restriction-enzyme site. Profiling fragments are separated on an acrylamide gel, which enables the detection of length polymorphisms that can be used directly for the purpose of genetic mapping. Marker sequences can be isolated from the gel in relatively high throughput and can be directly sequenced. Examples of the use of profiling markers to enrich existing genetic maps and to locate *R* gene clusters on these maps are provided by van der Linden *et al*. [24] in potato, tomato, barley and lettuce, Calenge *et al*. [25] in apple, Mantovani *et al.*[26] in durum wheat and Brugmans *et al*. [27] in potato. Other examples demonstrate how the profiling technique can be used in the absence of a reference map [28-31]. These studies used a bulked segregant approach to rapidly and efficiently identify profiling markers that locate close to the trait of interest.

In this study, we used a diploid mapping population from potato to validate the efficiency of multiple known and several new motif-directed profiling primers. A guideline is provided for the choice of primers to be used in different applications. Examples of new applications of the profiling technique, like comparative genomics and *R* gene fine mapping, are provided. Finally, we show that next-generation sequencing can be used to directly sequence profiling fragments and how these sequences can be used to identify the *R* gene family corresponding to late blight resistance.

## Results

### Coverage of *R* gene clusters using known NBS profiling primers

Known and novel NBS profiling primers were used to tag RGA sequences and successively locate clusters of RGA sequences in the genetic map of potato clone RH using the SHxRH diploid potato mapping population [27]. Using four different primers in combination with five different restriction enzymes, 732 markers from parent RH (Table 1) and a similar number of markers from the SH parent were generated. The RH markers were mapped to the Ultra-high Density (UHD) map [32] and, successively, grouped into bin ranges (Additional file 1: Table S1). A subset of the NBS profiling markers (209) was subjected to sequence analysis and 108 markers, deriving from 56 bin ranges, showed a high similarity to RGAs. Markers of 53 bin ranges showed the strongest homology to the CC type of RGAs (Figure 1; yellow bars). RGA cluster names (RHx.y) were assigned corresponding to a similar NBS profiling study by Brugmans *et al*. [27], where x is the chromosome number and y is the sequential number of the cluster on the chromosome. Fourteen RGA clusters were tagged again in our study, but 13 additional RGA clusters were also tagged (*RH3.1, RH4.1a, RH4.2a, RH5.2, RH5.4, RH6.3, RH6.4, RH7.1, RH8.1a, RH9.1a, RH9.1b, RH12.1* and *RH12.3*; indicated in italics in Figure 1). Compared to Brugmans *et al*. [27], a higher marker saturation was reached, probably due to the use of additional primers and enzymes. Also in a study performed by Bakker *et al*. [33], RGA clusters were anchored to the genetic map of RH. The RGA cluster positions in our study were very similar to the positions reported by Bakker *et al*. [33]. Only two additional RGA clusters were found (*RH3.1* and *RH12.1*) using the NBS primers (Figure 1, Additional file 1: Table S1).

**Table 1:** Number and sequence validation of markers generated in the SH*RH population using the different profiling primers and restriction enzyme combinations

**Primer**	***Alu*I**	***Hae*III**	***Mse*I**	***Rsa*I**	***Taq*I**	**Markers (#)**	**Tagged bin ranges (#)**	**Sequenced markers (#)**	on target* sequences (#)	on target**
										**bin ranges (#)**
NBS1	24	40	22	50	47	183	34	37	19	8
NBS2	51	36	56	43	50	236	29	37	14	6
NBS5a6	22	33	38	35	34	162	38	82	62	14
NBS9	22	25	18	40	46	151	29	53	13	6
total NBS	119	134	134	168	177	732	56	209	108	23
NBS13R	9	7	7	22	10	55	9	34	27	5
NBS15F	7	15	15	34	20	91	11	33	27	4
TIR300F	23	16	27	27	18	111	17	65	41	6
TIR300Fc	5	26	14	18	nd	63	9	40	32	4
TIR3R	16	35	11	12	15	89	12	38	20	3
TIR9256	13	13	29	23	13	91	13	49	22	2
TIRWCF	8	17	28	47	nd	100	17	49	26	6
total *N*-like	81	129	131	183	76	600	38	308	195	12
chcF2	14	3	9	4	nd	30	5	5	3	0
chcR1	5	1	12	9	nd	27	5	9	6	2
chcR2	8	5	9	15	nd	37	6	16	14	3
total *chc1*-like	27	9	30	28	0	94	9	30	23	3
PK1Fa	11	9	6	16	nd	42	12	20	13	4
PK1Fb	14	10	16	9	nd	49	16	20	11	8
PK3Fb	16	14	19	26	nd	75	16	42	11	6
PK4R1a	6	12	12	10	nd	40	13	16	4	2
total PK	47	45	53	61	0	206	35	98	39	19
total	274	317	348	440	253	1632	69	645	365	43

* Confirmed as on target when the sequence had significant homology to known R genes or RGAs.

** Confirmed as on target if the tagged bin range contained at least one on target sequenced marker.

# = numbers of.

**Figure 1:**
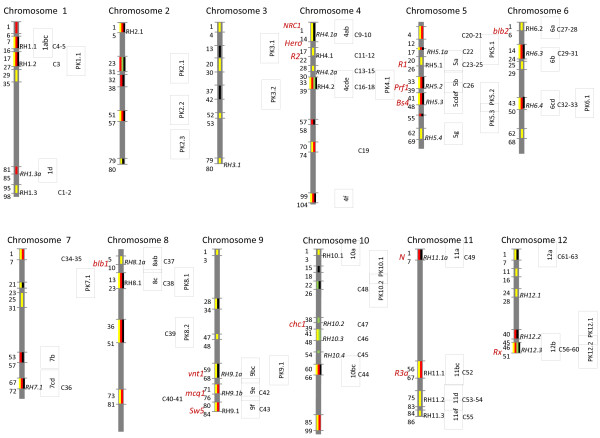
**Genetic map of RGA clusters in the potato genome.** The twelve chromosomes of potato are depicted. Bin ranges corresponding to the potato UHD map of RH are indicated by horizontal lines. Profiling markers in these bin ranges are identified with NBS, *N*-like, CDP^chc1^, or PK primers as indicated by yellow, red, green and black bars, respectively. The RHx.y label, on the right side of the chromosome cartoons, indicates RGA clusters as identified using NBS profiling by Brugmans *et al*. [27]. RGA clusters identified by profiling in this study are indicated as *RHx.y* labels (italic font). In the vertical text boxes, is the RGA cluster name given by Bakker et al. [33] In the second horizontal text boxes, the corresponding NLR cluster names, as provided by Jupe *et al*. [34] are given. In the second vertical text boxes, the names of clusters of confirmed PK sequences, as determined in this study, are provided. Positions of known *R* gene clusters, as derived from Bakker *et al*. [33] are indicated by red font.

### Cluster-directed profiling reveals additional RGA clusters

A striking observation in the sequence analysis of the NBS markers was that none of the sequences were significantly similar to the TIR class of RGAs. All RGA clusters tagged by the NBS primers had sequences with similarity to the CC type of RGAs (yellow highlight, Additional file 1: Table S1), and only in six RGA clusters sequences could not be assigned to either TIR or CC class of RGA (grey highlight, Additional file 1: Table S1). In addition, no similarity was found to the recently cloned *R* gene *Rpi-chc1*[35]. Attempts to broaden the target spectrum of the primers by including additional degeneracies were not successful because the specificity of amplification was rapidly lost and mainly off-target sequences were generated (data not shown). We tried to solve this apparent bias of the available NBS profiling primer set by adding several TIR derived or *N*-like profiling primers that were described in previous studies [30,36], and three new *Rpi-chc1* cluster-directed profiling (CDP) primers were designed. The NBS13R and NBS15F primers were derived from conserved parts of the NBS region of TIR class of RGAs. TIR300F, TIR300Fc, TIR3R, TIR9256 and TIRWCF were derived from conserved parts in the N-terminal TIR domains of known RGAs. Primers chcF2, chcR1, chcR2 were derived from conserved regions in the LRR region of the *Rpi-chc1* family [35]. The TIR-derived primers or *N*-like primers produced 600 markers that tagged 38 bin ranges (Table 1). Sequence analysis of 308 markers was successful, and 195 sequences indeed showed high homology to the TIR class of RGAs. These confirmed markers were located in 12 bin ranges that represent the TIR class of RGA clusters (Figure 1). When these clusters were compared to the results from Brugmans *et al*. [27] and Bakker *et al*. [33], nine (*RH1.3a, RH5.1a, RH5.2, RH5.3, RH6.3, RH6.4, RH7.1, RH11.1a and RH12.2*) and two (*RH5.1a and RH12.2*) new RGA clusters were identified, respectively (Figure 1, Additional file 1: Table S1). Using the *Rpi-chc1* primers, 94 markers were generated. Sequence analysis of 30 markers revealed three RGA clusters, all located on chromosome 10, harbouring 23 RGA sequences with homology to *Rpi-chc1*. None of these RGA clusters (*RH10.2, RH10.3 and RH10.4*) were tagged in previous studies [27,33].

### Map comparison and profiling marker saturation

The complete genome sequence of *S. phureja* [DM1-3 516R44 (DM), [37]] was recently analysed for the presence of NLR sequences [34]. The relative positions of *R* gene clusters on the chromosomes and sequence homology between the profiling markers in RH and the NLRs in the DM clusters were used to superimpose the *S. phureja* clusters on the RH map. Insufficient profiling markers and corresponding sequence data were available from chromosomes 2 and 3 to perform this comparative study. For the remaining ten chromosomes, the comparison of RGA locations revealed that the vast majority of the clusters were syntenic between both genomes (Figure 1), and 58 clusters could be matched between RH and DM. For six RH clusters, no equivalents were found in the DM genome and at least three DM clusters were not found in RH (Figure 1, Additional file 2: Table S2). For instance, the C40 and C41 clusters from DM contain sequences of the CNL6=*Rpi-blb1* family. No counterpart is found in RH in this study, nor in the study by Bakker *et al*. [33]. On the other hand, the RH12.2 cluster, which contains profiling markers with homology to *N*, is not identified in DM (Figure 1). This might reflect differences between the genomes. Alternatively, some *R* gene families (like the CNL3 family) may be targeted at low efficiency as was observed for the CNL7=*Rpi-chc1* family using the NBS primers. In addition, the assembly of the DM genome might still contain some gaps, especially at highly repetitive DNA sequences. Overall it can be concluded that by using a well-selected set of profiling primers a near complete coverage of NLR gene clusters can be achieved.

### Application of CDP markers for the purpose of fine mapping

**Figure 2:**
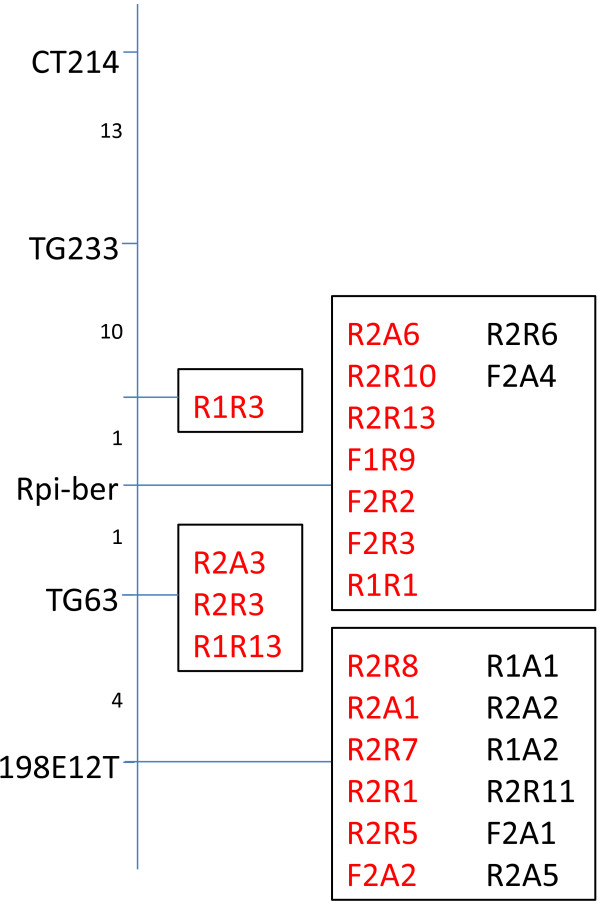
**Genetic map of the *Rpi-ber *gene.** Known CAPS markers are shown on the left side. On the right side, the CDP^chc1^ markers are shown. Numbers between horizontal lines represent the numbers of recombinants in an F1 population with 1,771 individuals. Marker names in red font are in *trans*-phase, while markers in black font are in *cis*-phase.

As described above, CDP using primers derived from *Rpi-chc1* (like) sequences resulted in multiple closely linked markers on chromosome 10 of potato clone RH. It is known that a late blight resistance gene from *Solanum berthaultii* is located on chromosome 10 [38,39]. *Rpi-chc1* CD profiling produced 53 markers that were segregating in a *S. berthaultii* population. Eight of these markers were linked *in cis*, and 17 were linked *in trans* to resistance. Twenty-eight markers were derived from the susceptible parent or were not linked to resistance. This showed that the *Rpi-chc1* CDP markers were highly specific and that the *S. berthaultii* late blight *R* gene is likely a homolog of *Rpi-chc1*. Also, the high number of linked markers that was found indicates that marker saturation was reached for this RGA cluster and that multiple paralogs from the cluster were possibly tagged. To confirm these hypotheses we pursued a fine mapping approach. From a set of 1,771 F1 seedlings, 25 plants were selected that had a recombination between the flanking markers of the *Rpi-ber* gene, TG63 and CT214 [38,39]. Indeed when the *Rpi-chc1-*like profiling markers were tested on this population, closely linked and even fully co-segregating markers were found (Figure 2). Fully co-segregating markers could potentially be located inside the *Rpi-ber* gene. In future research, these CDP markers will be highly instrumental for the map-based cloning of the *Rpi-ber* gene.

### PK profiling tags additional loci that might be involved in disease resistance

For *de novo* mapping of a new (resistance) trait, a marker technique is required that gives full genome coverage. Many marker techniques were shown to have a bias to certain parts of the genome resulting in marker clustering on one hand and blind spots on the other hand. As shown in the previous section, profiling with the NBS primers shows a severe bias towards a subset of RGA gene clusters. This bias could be partially resolved by using additional CDP primers. However, RGA sparse regions of the genome still remained unexposed. For de novo mapping of a resistance trait, this may be a serious drawback. Since it is known that besides the NLR type of *R* genes other types of *R* genes, such as protein kinases (PK) and receptor like protein kinases (RLK), govern resistance traits, a PK profiling strategy was pursued. Degenerate primers were designed based on alignments of PK sequences deriving from a very diverse set of species ranging from a monocot species like maize and rice to the dicots Arabidopsis, potato and tomato. In these genomes, more than 1,000 kinase-like sequences are found (data not shown) and indeed a high number of bands was found in Licor gels after PK profiling reactions (Additional 3: Figure S1); this is many more than observed in a typical NBS profiling gel. The number of polymorphic bands, however, was lower (Table 1). On average 13 PK markers per primer enzyme combination could be mapped to the RH map, while 36 NBS markers were mapped per primer enzyme combination. This suggests that the number of PK targets is higher in the potato genome, but the level of polymorphism in PK genes is lower as compared to NBS genes. About half of the PK marker sequences could be confirmed to potentially encode a protein kinase (confirmed on target sequences, Table 1). This frequency was comparable to the on-target frequency obtained with the NBS profiling markers. PK profiling, therefore, is a useful tool to tag PK genes in the genome. Nineteen bin ranges with confirmed PK markers were assigned with a PKx.y code (Figure 1). An additional 16 bin ranges contained unconfirmed PK markers (Table 1, Figure 1). PK markers could be generated on chromosomes and chromosome arms where NBS profiling markers were scarce or even absent (Additional file 1: Table S1). Using a combination of PK, NBS, CDP and *N*-like profiling, a total of 69 bin ranges were tagged. This suggests that a combined profiling approach covers most of the genome and will be a useful tool for the mapping of novel resistance traits. In addition to potato, PK profiling was also found to work in monocot species. In a population derived from a cross between *Avena strigosa* and *Avena wiestii*[40,41], PK profiling produced a large number of (clustered) markers distributed over the *Avena* genomes [42]. Interestingly, PK profiling markers were found in one of the two major loci for resistance to *Puccinia coronata*. Also in bread wheat, PK profiling markers were found to be associated with resistance to stripe rust (*Puccinia striiformis* f. sp. *tritici,* Sara Deszhetan, unpublished results).

### Next-generation profiling

**Figure 3:**
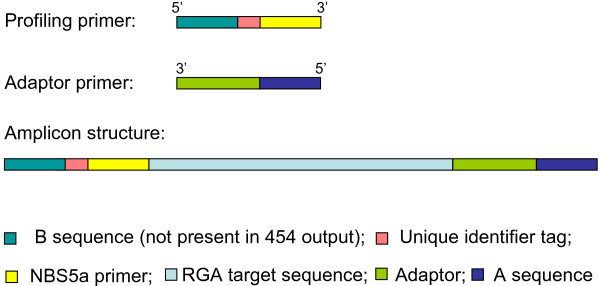
Schematic representation of next-generation profiling primers and amplicons.

Next-generation sequencing provides possibilities to simultaneously sequence pools of different amplicons. The combination of profiling with next-generation sequencing offers enormous advantages over the classical, gel-based way of profiling. We, therefore, designed a new experimental setup to produce profiling fragments for 454 sequencing. The genomic DNA of both parents and 44 F1 individuals of the SH*RH population was fragmented using mechanical shearing, and libraries of individuals were prepared by ligation of the profiling adaptors, that were extended with a 454-A-sequence at the 5' end (Figure 3), to the ends of mechanically sheared genomic DNA. As a basis for the next-generation profiling primer, we used the NBS5a primer that was extended at the 5’ side with a 454-B-sequence followed by a unique identifier (UID) tag resulting in 12 different NBS5a-next-generation profiling primers. The ligation libraries of the different individuals were converted into amplicon libraries using the 12 different NBS5a-next-generation profiling primers in combination with the next-generation adaptor primer. The amplicons had a predicted structure as depicted in Figure 3, and this structure was confirmed after cloning the fragments in *E coli* and sequencing 60 colonies (data not shown). Four pools of amplicons, derived from twelve individuals each, were made and sequenced in parallel in four quarter 454 GS-FLX reactions. Because sequencing started from the B primer, fused to NBS5a, all reads started at similar positions in the target RGAs. A total of 280,000 reads were produced but reads smaller than 150 nt, mainly primer dimers, were discarded. The remaining 239,000 reads were grouped per genotype based on the UID sequence. The UID sequence and the NBS5a sequence were then trimmed off from the 5’ end, resulting in reads with an average length of 166 nt. Samples deriving from the parental plants SH and RH were included twice in this experiment and were further analysed to estimate the sequence depth. In the parental samples, an average of 4,640 reads was found with 2,773 reads found more than once, leaving an average of 1,867 unique sequences per sample (Additional file 3: Table S3) suggesting an average depth of 2.5x. When the reads were compared among duplicate samples, we found an average of 542 unique sequences suggesting an average depth of 6x. This discrepancy might be explained by the occurrence of sample-specific PCR errors (like homo-polymer errors) that inflate the number of unique sequences.

To verify the efficiency to target RGA sequences, all 239,000 reads were BLASTed against a set of 34 known *R* gene sequences from *Solanaceae*. As shown in Table 2, the majority of the reads (140,823) showed BLAST E-values below 1*e^-20^, which corresponds to more than 80% identity for sequences of this size. This confirmed that our approach was indeed tagging RGAs from the potato genomes with a very high efficiency. In order to further compare the next-generation profiling approach to the gel-based profiling, the 454 reads were grouped by homology to *R* gene families. The reads with an E-value higher than 1*e^-20^ were disregarded for this study. For the remaining reads, the hit with the lowest E-value was listed. Table 2 shows that the *Hero*, *Mi1.2/Rpi-blb2* and *I-2/R3a/R3b* gene families were represented most in the next-generation profiling reads. Members of these families were probably preferentially amplified in the profiling reaction, while members of other large families were either less efficiently amplified or target sequences were less abundant. RGAs with homology to *Hero*, *Mi1.2/Rpi-blb2* and *I-2/R3a/R3b* mapped in bin ranges RH4 bin1-14, RH6 bin1-6, and RH11 bin84-86, respectively. As is shown in Additional file 1: Table S1, these bin ranges indeed contain the majority of the gel-based NBS5a profiling markers. In addition, the notion that NBS5a did not produce any markers in the *N*-like cluster on chromosome 11 (*RH11.1a*) was confirmed using this next-generation profiling approach, only two reads had homology to *N*. In contrast, the *Rpi-chc1* clusters (*RH10.2, 3, 4*) on chromosome 10 were tagged only once using the gel-based profiling approach, while the next-generation profiling approach produced almost 700 reads with highest similarity to *Rpi-chc1*. The next-generation profiling approach might be less biased than gel-based profiling, possibly due to the mechanical DNA fragmentation rather than the enzymatic fragmentation used in gel-based profiling. However, new biases might be introduced since RGAs from *Tm2* clusters on chromosome 9 (RH9 bin47-68) were sequenced with low frequency using next-generation profiling, while three gel-based profiling markers were found in this genomic area (*RH9.1a*, Figure 1, Additional file 1: Table S1).

**Table 2:** 454 sequence reads in the SH*RH population

**complete reads (#)**		bulk* specific K-mers (#)								***R *gene family with highest homology**
**BR**	**BS**	**BR**	**BS**	**BR**	**BS**	**BR**	**BS**	**BR**	**BS**	
		>= 2		>= 7		>= 12		>= 15		
17990	29516	128	256	0	20	0	1	0	0	*Mi-1.2/Rpi-blb2*
14848	28044	68	298	0	21	0	0	0	0	*Hero*
13487	23565	172	439	37	70	18	9	3	3	*I-2/R3a/R3b*
1427	2320	81	150	0	3	0	0	0	0	*RB/Rpi-blb1/Rpi-pta1/Rpi-sto1*
1228	2520	53	223	0	0	0	0	0	0	*R1*
1230	2011	64	99	0	0	0	0	0	0	*Rpi-blb3/R2/Rpi-abpt1*
277	516	14	29	0	0	0	0	0	0	*NRC1*
293	402	6	146	0	33	0	0	0	0	*Rpi-chc1*
209	362	0	25	0	2	0	0	0	0	*Sw-5a*
207	283	5	10	0	3	0	0	0	0	*Rx/Gpa2*
43	43	0	0	0	0	0	0	0	0	*Prf1*
22	31	0	0	0	0	0	0	0	0	*Tm-2/Rpi-vnt1*
1	1	0	0	0	0	0	0	0	0	*N*
51240	89583									total (E-value < e-20)**
88475	150513	591	1675	37	152	18	10	3	3	total

>=n: K-mers that occurred more than n times were counted.

* Individuals were grouped in resistant (*R3a* and *R3b*) and susceptible bulks (BR and BS).

** E-values were obtained after BLAST analysis against a database of 34 selected *R* genes.

# numbers of.

### Application of next-generation profiling for the identification of *R* gene families involved in disease resistance

RGA fragments generated using gel-based profiling can readily be used for genetic mapping based on the absence or presence of a band of a specific size. Due to the insufficient coverage in this experiment, presence and absence scoring of a particular sequence was not feasible. We, therefore, studied whether a bulked segregant approach was feasible to identify candidate genes or candidate gene families. Genotype SH contains the closely linked *R3a* and *R3b* genes that confer resistance to late blight, and these genes cause the late blight resistance segregating in the SH*RH population [18,43]. We grouped the next-generation profiling sequences from 14 resistant and 29 susceptible individuals in a resistant (BR) and a susceptible bulk (BS), respectively. In order to rule out an effect of differences in sequence lengths, the reads were first size trimmed, and only the 100 bp after the NBS5a primer were maintained. Bulk-specific, 100-bp sequences that occurred at least 2, 7, or 12 times were BLASTed against the 34 *R* gene set and the highest BLAST hits were retrieved. Unfortunately, *R* genes from multiple chromosomes were found, which showed that some sequences in our set were causing an artificial bulk specificity. In order to exclude sequence errors, the 100-nt tags were split into K-mers of 31 nt. Again bulk-specific sequences were identified and unique (n=1) sequences were discarded. This way 2,266, 189, and 28 bulk-specific K-mers that occurred at least 2, 7, or 12 times, respectively (Table 2) were identified. These K-mers were BLASTed against the 34 *R* gene set. All of the 37 BR-specific K-mers and half of the BS-specific K-mers that occurred at least 7 times showed the highest similarity to members of the *R3* gene family. The identification of *R3* sequences validated our approach since clusters of *R3*-like sequences are present both *in cis* (BR) and *in trans* (BS) phase to late blight resistance caused by *R3a* and *R3b*[18]. Remarkably, half of the BS-specific K-mers that occurred at least 7 times had homology to other RGA families, which are regarded as unspecific. When the threshold was raised to n = 12, only one unspecific BS sequence remained, and further elevation to n = 15 even eliminated the unspecific sequences. In the current experiment, the coverage was relatively low, which resulted also in a loss of 25 of the 28 *R3*-like sequences with a further increase of the threshold. Altogether, these results show that next-generation profiling combined with bulked segregant analysis is a potential tool to identify gene families involved in resistance traits.

## Discussion

### An evaluation of profiling primers for targeting diverse parts of the genome

Profiling techniques have been used to efficiently target NLR genes, RGAs and other gene families. In this study, we tested additional NLR primer enzyme combinations resulting in the tagging of additional RGAs in potato as compared to a previous profiling study [27]. Furthermore, it is specified how efficient the different profiling primers are in tagging the different RGA clusters. Comparisons to the results from the study of Bakker *et al*. [33] showed that some TIR-NLR or N-like clusters are underrepresented or even absent with a first set of primers. This was confirmed by next-generation profiling with the NBS5a primer. The absence of N-like targets could be bypassed using (degenerate) primers derived from N-like sequences. In addition, other underrepresented clusters, like the *Rpi-chc1* cluster on chromosome 10, could be efficiently targeted using dedicated primers. This approach is referred to as cluster-directed profiling (CDP). Using a diverse set of NBS, *N*-like and *Rpi-chc1*-like primers (NLR primers), the vast majority of NLR clusters could be targeted (NLR profiling). Compared to the NLR clusters in the DM genome [34], a few clusters were still not tagged by profiling of clone RH. Additional CDP primers could be designed to tag the sequences in these remaining clusters for dedicated purposes in follow-up studies. Alternatively, the discrepancy between our profiling results and the results presented by Jupe *et al*. [34] might be caused by differential NLR clustering in the RH and DM genomes.

The profiling markers in this study could be mapped to the UHD map of RH [32]. Profiling using NLR primers is not suitable for *de novo* mapping because of the clustering of the NLRs leaving large regions of the genome untagged, which will result in incomplete genetic maps (Table 2). The PK profiling technique was shown to tag 'blind spots’ in the NLR profiling map. By targeting combinations of additional gene families or repeated sequences such as transposons [44,45], a genome wide coverage sufficient for *de novo* mapping can be achieved.

### An evaluation of profiling primers for targeting genes involved in disease resistance

Many studies have been published that describe the localisation of *R* genes in genetic maps using profiling markers [28-31,36]. Here, we describe the use of CDP markers for fine mapping of the *Rpi-ber* late blight resistance gene. This is a novel application of the profiling technique through which many different paralogous NLRs in a cluster can be efficiently tagged. These markers can be readily used in the process of *R* gene cloning to select or eliminate candidate RGAs within a cluster. It must, however, be realised that mixed clusters are present in the genome [34], which would complicate a CDP approach. Also many unclustered RGA sequences, whose biological function is not clear yet, have been reported in the DM genome. It is unlikely that these unclustered RGAs will be efficiently tagged by NBS, let alone CDP profiling.

Another example of how profiling markers can be used to tag resistance traits in monocots was provided by Sanz *et al*. [42] who identified PK profiling markers that were associated with rust resistance in oat. In dicots, only a few resistance traits were shown to be governed by PK genes [13-15]. The role of dicot RLK genes seems to be limited to accessory components of disease resistance (reviewed by [46]) or to the perception of pathogen-associated molecular patterns that provide only minor levels of disease resistance (reviewed by [47]). In monocots, RLKs seem to play a more prominent role in resistance to bacterial pathogens. It remains, therefore, elusive whether the PK markers associated with rust resistance in wheat and oats are located in (the clusters of) the genes providing disease resistance.

In the absence of a reference map, the sequence of a profiling marker can give a good indication about the chromosomal position of the target sequence [28-31,36]. However, this indication can also be misleading. Jo *et al*. [31] found that the sequence of a NBS profile marker that was linked to the *R8* late blight resistance gene had homology to *Hero*, a nematode *R* gene that was located on chromosome 4. Closer study revealed the presence of *Hero*-like sequences on chromosome 9 as well. In this study, we showed by next-generation profiling that the chromosomal location of the *R3a* and *R3b* genes could be reiterated by bulk-specific sequences. However, in the susceptible bulk, which is enriched for the trans phase of the *R3* resistance genes, both R3 sequences and *Rpi-chc1*-related sequences were found. This could represent an artefact caused by the low coverage, but it might also indicate an unexpected genomic position of RGA sequences, possibly caused by recent translocations.

### An evaluation and future of next-generation profiling

Profiling is a very efficient technique to generate markers for a wide range of purposes, as elaborated in this study. However, it is also relatively laborious. Polyacrylamide gel electrophoresis must be used to detect small differences in the molecular weights of the PCR fragments. Also the identification of the markers and the scoring of the markers of many individual samples is time consuming and prone to errors. Moreover, sequence analysis of the marker bands requires isolation from the acrylamide gel, and sometimes the bands require cloning before sequencing can be properly performed. We resolved several of these issues by designing and using a next-generation, sequencing-based protocol that slightly deviates from the classical, gel-based, profiling protocol. Fractionation of the genomic DNA by restriction enzymes was replaced by mechanical fractionation because restriction enzymes may introduce a bias towards a subset of fragments. Amplicons were sequenced from the B primer that was adjacent to the NBS5a sequence. In this way, the sequence reads were anchored to comparable sites in the target sequences, which allows the alignment of the reads directly without the need of a prior assembly step. This is a great advantage since correct assembly of sequences derived from paralogs and alleles with high sequence similarity is extremely challenging. Trimming of the reads at the A side and partitioning into K-mers were additional sequence-processing steps necessary to allow the detection of BR- and BS-specific sequences derived from the *R3* cluster. Since the resistance to late blight in the SH*RH population is conferred by *R3b*, it was concluded that next-generation profiling efficiently predicts the family to which an *R* gene belongs. Future mapping of new (late blight) *R* genes can be significantly sped up using this new approach. In future studies, individuals can be pooled prior to sequencing. In this way, multiple populations could be studied using a single 454 run. Moreover, we showed that the first 100 nt of the reads, and even less, were sufficient to distinguish the RGA families they derived from. This means that next-generation profiling could also be performed on other next-generation sequencing platforms that produce shorter but more reads; this provides higher coverage and/or more populations that can be sequenced simultaneously.

One application of classical gel-based profiling is the genome-wide mapping of NLR sequences or sequences of any other gene family. We pursued a “genotype by sequence” approach with the presented next-generation profiling sequences. Although the sequences deriving from the different individuals could be efficiently separated using the UID tags, unfortunately, the estimated sequence depth of 2.5 till 6* was too low. It is estimated that a mapping approach will be feasible if at least a ten-fold higher coverage can be reached, which is required to distinguish between the presence or absence of the newly identified sequences in different individuals. Future studies using the latest 454 technology would give a higher coverage but still it would not be sufficient for mapping NBS sequence reads. The superior sequence depth of Solexa technology would be required. In this way, even a larger number of individuals can be included to allow more accurate mapping. In order to reach full genome coverage, a mixture of profiling primers could be used, and the acquired marker sequences can be combined for (*de novo*) mapping. The distribution of gel-based profiling markers, obtained by the primers in Table 2, could serve here as a guide to select primers for next-generation profiling approaches. In addition, genome-wide mapping or more focussed CDP studies can be pursued.

## Methods

### Plant material and DNA isolation

A total of 41 F1 progeny and both parental plants were selected from the diploid SH*RH (SH83-92-488*RH89-039-16) population that was used for generating the UHD potato map as described by [32] (http://www.plantbreeding.nl/Projects/UHD/index.html). Among these 43 plants, 14 individuals were resistant to *P. infestans* isolate 89148–9 (genotypes: #27, #34, #130, #138, #164, #178, 23, 31, 38, 51, 58, 65, 83 and SH83-92-488) and 29 individuals were susceptible ( #11, #49, #51, #53, #57, #59, #64, #137, #157, #159, #169, #179, #190, 6, 11, 17, 29, 33, 35, 39, 48, 54, 60, 61, 63, 64, 86, 89 and RH89-039-16). In addition, 29 F1 recombinants were selected from a *S. berthaultii* population (n = 1771; G254*94-2031), which had been identified as a source for resistance to *P. infestans*[39]. For molecular genetic analyses, genomic DNA was isolated from meristematic leaf material of 3–6 week old greenhouse grown plants as described by Fulton *et al*. [48].

### Motif-directed and cluster-directed profiling

Motif-directed Profiling was carried out on genomic DNA as described by van der Linden *et al*. [24]. The restriction enzymes *Alu*I, *Mse*I, *Rsa*I, *Hae*III and *Taq*I were used for digestion of genomic DNA. Sequences of the degenerate primers used for the amplification of specific fragments are shown in Table 3. New profiling primers were designed based on reverse translated protein sequence alignments of specific conserved blocks of amino acids (motifs) in NLR or PK protein sequences. PCR products were separated on a 6% polyacrylamide gel, and the individual fragments were visualised by fluorescence in a Li-Cor machine (Additional file 4: Figure S1). Polymorphic bands observed were scored for their presence/absence in the progenies. In the SH*RH population, the relative genetic positions of each candidate RGA marker was calculated using maximum likelihood mapping [32]. In the UHD map, the genetic bins are defined by single recombination events and correspond to a genetic distance of 0.8 cM. Using the BINMAP-plus application (Borm, unpublished), 1,641 markers could be reliably (LOD > 4) mapped to the UHD map. Loci defined by a single marker with a LOD < 4 were ignored. Also marker loci spanning more than 10 bins were ignored for cluster definition.

**Table 3:** Profiling primer sequences and annealing temperatures

**Primer name**	**Primer sequence**	**Ta**	**Reference**
Pk1Fa	GGyTTTGGwrinGTyTACAAAGG	52	[42]/this study
PK1Fb	GGyTTTGGwrinGTyTAtAAGGG	52	[42]/this study
PK4R1a	yykrGCyAGyCCAAAATC	54	[42]/this study
PK3Fb	rTyGTrCATAGrGACATCAA	54	[42]/this study
NBS1	GCiArwGTwGTyTTiCCyrAiCC	55	[49]
NBS2	GTwGTyTTiCCyrAiCCissCAT	60	[24]
NBS5a6	yyTkrThGTmiTkGATGAyrTiTGG	55	[49]
NBS9	TGTGGAGGrTTACCTCTAGC	55	[24]
chcR1	TGGmCkrAGAAAmCCTTCwGCCATC	66	This study
chcR2	CCwArrCCwsCCATwCCyACTAT	62	This study
chcF2	CTACCAmkyCGAsArAcAGATTCC	64	This study
NBS13R	AAGAArCATGCdATATCTArAAATAT	55	[30]
NBS15F	ATGCATGAyTTrATwvAAGAbATGGG	55	[30]
TIR300Fc	TAGTrAAGATyATGGAATGCA	55	This study
TIR300F	nTAGTrAAGAyATGGAATGC	55	[30]
TIR9256	ATGGCATCTTCTTCTTCTTCTTTTGCG	55	[30]
TIR3R	CTTCACTAnTTCATyCAAGCACC	55	[30]
TIRWCF	AGAATTAyrCiACrTCiAGrTGGTG	60	This study
Adapt-upper	ACTCGATTCTCAACCCGAAAGTATAGATCCCA		[24]
Adapt-lower blunt	TGGGATCTATACTT-NH2		This study
TG233F	CATGCCTTTTTCTTGGGATG		This study
TG233R	TGGAACCCCTTTAACTGTGC		This study
198E12T-F	GACTCTGCCGTGATTGCTGAA		This study
198E12T-R	CACCGGGAAGACGCTGTTT		This study
Aseq primer	GCCTCCCTCGCGCCATCAG		Roche
Bseq primer	GCCTTGCCAGCCCGCTCAG		Roche
454Adpt Primer	GCCTCCCTCGCGCCATCAGGTTTACTCGATTCTCAACCCGAAAG		This study
454B01-NBS5a	GCCTTGCCAGCCCGCTCAGACGAGTGCGT yyTkrThGTmiTkGATGAYGTiTGG		This study
454B02-NBS5a	GCCTTGCCAGCCCGCTCAGACGCTCGACA yyTkrThGTmiTkGATGAYGTiTGG		This study
454B03-NBS5a	GCCTTGCCAGCCCGCTCAGAGACGCACTC yyTkrThGTmiTkGATGAYGTiTGG		This study
454B04-NBS5a	GCCTTGCCAGCCCGCTCAGAGCACTGTAG yyTkrThGTmiTkGATGAYGTiTGG		This study
454B05-NBS5a	GCCTTGCCAGCCCGCTCAGATCAGACACG yyTkrThGTmiTkGATGAYGTiTGG		This study
454B06-NBS5a	GCCTTGCCAGCCCGCTCAGATATCGCGAG yyTkrThGTmiTkGATGAYGTiTGG		This study
454B07-NBS5a	GCCTTGCCAGCCCGCTCAGCGTGTCTCTA yyTkrThGTmiTkGATGAYGTiTGG		This study
454B08-NBS5a	GCCTTGCCAGCCCGCTCAGCTCGCGTGTC yyTkrThGTmiTkGATGAYGTiTGG		This study
454B09-NBS5a	GCCTTGCCAGCCCGCTCAGTAGTATCAGC yyTkrThGTmiTkGATGAYGTiTGG		This study
454B10-NBS5a	GCCTTGCCAGCCCGCTCAGTCTCTATGCG yyTkrThGTmiTkGATGAYGTiTGG		This study
454B11-NBS5a	GCCTTGCCAGCCCGCTCAGTGATACGTCT yyTkrThGTmiTkGATGAYGTiTGG		This study
454B12-NBS5a	GCCTTGCCAGCCCGCTCAGTACTGAGCTA yyTkrThGTmiTkGATGAYGTiTGG		This study

Ta: annealing temperature.

### Isolation and sequence analysis of profiling fragments

Polymorphic bands were cut out of polyacrylamide gels using a scalpel knife, eluted in 100 μl of TE, put at 97°C for 5 min, and reamplified with the specific primer and the adapter primer. PCR products were checked on agarose gels. Fragments were directly sequenced using the adapter primer as a sequencing primer. Sequencing was carried out with the BigDye Terminator kit and an ABI 3700 automated sequencer from Applied Biosystems (USA). Sequences were identified by comparison with entries in the public protein and nucleotide databases using BLASTX and tBLASTX programs (Altschul *et al*., [50]).

### Next-generation profiling

The construction of NBS amplicon libraries was performed according to the profiling protocol that was described by [24]. The protocol was modified for parallel sequencing of amplicon pools from multiple samples. These modifications were based on the GS FLX Shotgun Library Method Manual and the GS FLX Amplicon DNA Library Preparation Method Manual [51]. Briefly, 5 μg of genomic DNA was nebulized into fragments of 300 to 2,000 bp, fragments were blunt-end repaired using T4 polymerase, and adaptors were ligated to the end of the fragments. For the amplification of putative NBS fragments, 12 fusion primers were synthesised (Isogen Life Science) consisting of the 454 A adaptor, a 10-bp validated barcode (Roche) and the NBS5A primer sequence (CTGATGGCGCGAGGGAGGCxxxxxxxxxxYYTKRTHGTMITKGATGAYGTITGG). The universal primer consisted of the 454 B adaptor and the adaptor primer sequence (GCCTCCCTCGCGCCATCAGGTTTACTCGATTCTCAACCCGAAAG). Amplifications were performed in a total of 50 μl with 20 pmol of each primer, 200 μM dNTPs, 0.4 U HotStarTaq (Qiagen), and 5.0 μl of HotStarTaq PCR buffer on a PTC-200 thermocycler (MJ Research, Waltham, Mass., USA), using the following cycling program: 35 cycles of 30 s at 95°C, 1 min 40 s at 55°C and 2 min at 72°C. After amplification, the products were purified and size fractioned (> ~300 bp) using AMPure beads (Agencourt) and quantified using an Agilent 2100 BioAnalyzer. Resultant fragments ranges were between 300–1,000 bp with the majority of the fragments between 400–700 bp and an average of 122 nmol/μl. Four amplicon pools were composed by equimolar pooling of the NBS fragments of 12 samples. The DNA of the parents was processed in duplicate. Emulsion PCR and sequencing was carried out according to the standard Roche/454 GS20 routine.

## Abbreviations

R gene: Resistance gene; RGA: Resistance gene analog; CDP: Cluster-directed profiling; NBS: Nucleotide binding site; LRR: Leucine-rich repeat; NLR: Nucleotide binding leucine-rich repeat proteins; RLP: Receptor-like proteins; RLK: Receptor-like kinases; PK: Protein kinases; TIR: Toll/interleukin-1 receptor/R gene domain; CC: Coiled-coil; UID tag: Unique identifier tag; BR: Resistant bulk; BS: Susceptible bulk.

## Competing interests

The authors declare that they have no competing interests.

## Authors’ contributions

JV wrote the manuscript and was responsible for the experimental design. SD performed the TIR, and PK profiling in SH*RH and analysed the data. DE performed the next-generation profiling and data analysis. MA performed the *Rpi-ber* fine mapping. MS performed the PK profiling in oats. WV performed the initial bulked segregant analysis of the next-generation profiling results. EV initiated the TIR profiling studies. GVDL performed the NBS profiling in SH*RH and initiated the next-generation profiling project. All authors read and approved the final manuscript.

## Supplementary Material

Additional file 1: Table S1 Mapping of the profiling markers produced by the indicated profiling primers in defined RH bin ranges and comparison of bin ranges to published RGA clusters. Red highlights: Bin range containing at least one marker that was confirmed, by sequence analysis, to locate in a TNL gene. Yellow highlights: Bin range containing at least one marker that was confirmed, by sequence analysis, to locate in a CNL gene. Grey highlights: Bin range containing at least one marker that was confirmed, by sequence analysis, to locate in a NLR gene. It could not be deduced if the genes belonged to the CNL or TNL class. Orange highlights: Bin range containing both markers in CNL or TNL genes as determined by sequence analysis. Blue highlights: Bin range containing at least one marker that was confirmed, by sequence analysis, to locate in a PK gene. * RGA cluster names were extended according to the nomenclature of Brugmans *et al*. [27]. Newly identified clusters are marked using italic font. ** RGA cluster names according to the nomenclature of Bakker *et al*. Red, yellow and orange shade colours indicate TIR, non TIR, or mixed cluster, respectively, as described by Bakker *et al*. [33]. *** RGA cluster names according to Jupe *et al*. [34] Red, yellow and orange shade colours indicate TIR, non-TIR, or mixed cluster respectively as described by Jupe *et al*. [34]. **** *R* gene families were assigned using a consensus from the current and previous studies [27,33,34]. Click here for file

Additional file 2: Table S2 Discrepancies between RGA clusters from RH and DM. Click here for file

Additional file 3: Table S3 Identification of unique and identical sequences within samples and among duplicate samples. Click here for file

Additional file 4: Figure S1 Example of comparable parts of two Li-Cor gels containing TIRWC-F primer *Rsa*I and PK1Fb primer combined with *Mse*I. Arrows indicate the positions of several polymorphic bands that were scored and mapped. Overall, the number of both polymorphic and monomorphic bands produced by PK profiling primers was lower as compared to TIR and NBS profiling markers. Click here for file
