# Supplementary material for: Novel applications of motif-directed profiling to identify disease resistance genes in plants
Source: Plant Methods. 2013 Oct 7;9:37. doi: 10.1186/1746-4811-9-37 (PMC3853995; doi:10.1186/1746-4811-9-37)
Supplement: Additional file 1: Table S1 — Mapping of the profiling markers produced by the indicated profiling primers in defined RH bin ranges and comparison of bin ranges to published RGA clusters. Red highlights: Bin range containing at least one marker that was confirmed, by sequence analysis, to locate in a TNL gene. Yellow highlights: Bin range containing at least one marker that was confirmed, by sequence analysis, to locate in a CNL gene. Grey highlights: Bin range containing at least one marker that was confirmed, by sequence analysis, to locate in a NLR gene. It could not be deduced if the genes belonged to the CNL or TNL class. Orange highlights: Bin range containing both markers in CNL or TNL genes as determined by sequence analysis. Blue highlights: Bin range containing at least one marker that was confirmed, by sequence analysis, to locate in a PK gene. * RGA cluster names were extended according to the nomenclature of Brugmans et al. [27]. Newly identified clusters are marked using italic font. ** RGA cluster names according to the nomenclature of Bakker et al. Red, yellow and orange shade colours indicate TIR, non TIR, or mixed cluster, respectively, as described by Bakker et al. [33]. *** RGA cluster names according to Jupe et al. [34] Red, yellow and orange shade colours indicate TIR, non-TIR, or mixed cluster respectively as described by Jupe et al. [34]. **** R gene families were assigned using a consensus from the current and previous studies [27,33,34]. [file 1746-4811-9-37-S1.pdf]

	Chromosome 1						Chromosome 2				
BIN	1-4	7-16	17-27	29-35	81-85	95-98	1-5	23-31	32-38	51-57	79-80
NBS1	0	9	0	2	0	2	2	0	0	0	1
NBS2	0	7	0	1	0	1	0	0	0	1	1
NBS5a6	0	3	1	1	0	0	2	0	0	0	0
NBS9	0	10	4	0	0	0	3	0	0	0	0
NBS13R	0	7	1	0	1	0	0	0	0	0	0
NBS15F	0	3	1	0	2	0	0	0	0	0	0
TIR300F	0	9	0	0	0	0	0	0	0	1	0
TIR300Fc	0	8	0	0	0	0	0	2	0	0	0
TIR3R	0	8	0	0	0	0	0	0	0	0	0
TIR9256	0	10	2	0	0	0	2	0	1	0	0
TIRWCF	2	14	3	0	2	0	1	0	0	0	0
chcF2	0	0	0	0	0	0	0	0	0	0	0
chcR1	0	0	0	0	0	0	0	2	0	0	0
chcR2	0	0	0	0	0	0	0	0	0	0	0
PK1Fa	0	0	2	0	0	0	2	0	1	0	0
PK1Fb	0	1	1	0	0	0	0	2	0	1	4
PK3Fb	1	2	2	0	0	0	0	1	0	0	0
PK4R1a	0	4	1	0	0	0	0	0	0	0	2
Brugmans & this study*		RH1.1	RH1.2		*RH1.3a*	RH1.3	RH2.1				
Bakker**		1abc			1d						
Jupe***		C4-5	C3			C1-2					
R gene families****		N, Bs4	N, Bs4		N, Bs4		Put. RGA				

	Chromosome 3						Chromosome 4						
BIN	1-4	13	20-30	37-42	52-53	79-80	1-14	17-22	28-30	33-39	57-58	70-74	99-104
NBS1	2	0	0	0	0	3	2	2	5	5	0	0	0
NBS2	0	0	0	0	1	2	2	16	1	5	0	0	0
NBS5a6	2	0	0	0	0	0	6	0	8	5	0	1	0
NBS9	0	0	2	0	1	0	0	2	1	1	0	0	1
NBS13R	0	0	0	0	0	0	1	0	0	0	0	0	0
NBS15F	0	0	0	0	0	0	0	0	0	0	0	0	0
TIR300F	0	0	0	0	0	1	0	0	0	3	0	1	1
TIR300Fc	0	0	0	0	0	0	0	1	0	0	1	0	0
TIR3R	0	0	0	0	0	0	0	0	0	5	2	0	0
TIR9256	0	0	0	1	0	0	0	0	0	0	0	0	0
TIRWCF	0	0	0	0	0	0	0	0	0	0	0	1	0
chcF2	0	0	0	0	0	0	0	0	0	0	0	0	0
chcR1	0	0	0	0	0	0	0	1	0	0	0	0	0
chcR2	0	0	0	0	0	0	0	0	0	1	0	0	0
PK1Fa	0	7	0	0	0	0	0	0	0	1	1	0	0
PK1Fb	0	0	0	1	0	0	1	0	0	1	0	0	0
PK3Fb	0	0	0	0	0	0	0	0	0	8	0	0	0
PK4R1a	0	0	0	1	0	0	0	0	0	1	0	0	1
Brugmans & this study*						*RH3.1*	*RH4.1a*	RH4.1	*RH4.2a*	RH4.2			
Bakker**							4ab			4cd			4f
Jupe***							C9-10	C11-12	C13-15	C16-18		C19	
R gene families****						R3a, R3b	**Hero, Nrc1**	**R2**	put. RGA	R3a, R3b			

	Chromosome 5							Chromosome 6				
BIN	4-12	17	20-26	33-39	41-48	55	62-69	1-6	14-24	25-29	43-50	62-68
NBS1	1	0	1	0	2	0	2	1	2	0	0	0
NBS2	10	0	4	0	8	0	0	10	1	0	9	1
NBS5a6	0	1	6	1	6	0	3	13	3	2	3	1
NBS9	1	0	0	0	4	0	0	0	8	1	1	0
NBS13R	0	0	0	0	0	0	0	0	3	0	1	0
NBS15F	0	0	0	1	2	0	0	0	9	0	3	0
TIR300F	0	1	0	0	4	0	0	0	2	0	0	0
TIR300Fc	0	0	0	0	2	0	0	0	0	0	0	0
TIR3R	1	0	0	0	3	0	0	0	5	0	0	0
TIR9256	0	0	0	0	1	0	0	0	0	0	2	0
TIRWCF	1	0	0	0	3	1	0	0	0	0	0	0
chcF2	0	0	0	0	0	0	0	0	0	0	0	0
chcR1	0	0	0	0	0	0	0	0	0	0	0	0
chcR2	0	0	0	0	0	0	0	0	0	0	0	0
PK1Fa	0	0	0	0	3	1	0	0	0	0	0	0
PK1Fb	0	1	0	0	1	0	0	0	0	0	0	0
PK3Fb	0	0	0	0	1	0	0	0	5	0	1	0
PK4R1a	0	0	0	1	0	0	0	0	0	0	0	0
Brugmans & this study*		*RH5.1a*	RH5.1	*RH5.2*	*RH5.3*		*RH5.4*	RH6.2	*RH6.3*		*RH6.4*	
Bakker**			5a	5b	5cdef		5g	6a	6b		6cd	
Jupe***	C20-21	C22	C23-25	C26				C27-28	C29-31		C32-33	
R gene families****		N, Bs4	**R1**	**Prf1**, N	N, **Bs4**		R3a, R3b	**Rpi-blb2**	N, Bs4		N, R3a, R3b	

	Chromosome 7					Chromosome 8				Chromosome 9						
BIN	1-7	21-23	25-31	53-57	67-72	5-10	13-23	36-51	73-81	1-3	28-34	47-48	59-68	71-76	80-81	84
NBS1	1	1	0	0	4	0	1	1	1	0	7	0	0	2	1	0
NBS2	0	0	0	0	7	1	2	0	2	0	1	0	0	0	17	0
NBS5a6	1	0	1	0	3	2	2	1	0	0	2	2	3	0	1	2
NBS9	1	0	1	0	0	2	0	0	0	3	1	0	1	3	5	0
NBS13R	0	0	0	0	0	0	0	0	1	0	0	0	0	0	0	0
NBS15F	0	0	0	0	0	0	3	0	0	0	0	0	0	0	0	0
TIR300F	0	0	0	1	3	0	4	0	0	0	0	0	0	0	0	1
TIR300Fc	0	0	0	0	0	0	1	0	0	0	0	0	0	0	0	0
TIR3R	0	0	0	0	1	0	0	0	1	0	0	0	0	0	0	0
TIR9256	0	0	0	0	1	0	5	2	0	0	0	0	0	0	0	0
TIRWCF	2	0	0	1	2	0	1	0	0	0	0	0	0	1	0	0
chcF2	0	0	0	0	1	0	0	0	0	0	0	0	0	0	0	0
chcR1	0	0	0	0	0	0	0	0	0	0	0	0	0	0	0	0
chcR2	0	0	0	0	0	0	0	0	0	0	0	0	0	0	0	0
PK1Fa	0	0	0	0	0	0	0	1	0	0	0	0	0	0	0	0
PK1Fb	0	0	0	0	1	0	1	2	0	0	1	0	0	0	0	0
PK3Fb	0	0	0	1	1	0	3	0	0	0	0	0	2	0	0	0
PK4R1a	0	1	0	0	0	1	0	1	0	0	0	0	0	0	0	0
Brugmans & this study*					*RH7.1*	*RH8.1a*	RH8.1						*RH9.1a*	*RH9.1b*	RH9.1	
Bakker**				7b	7cd	8ab	8c				9bc	9e			9f	
Jupe***	C34-35				C36	C37	C38	C39	C40-41					C42	C43	
R gene families****					N, Bs4	Rpi-blb1	N, Bs4						**Rpi-vnt1**	**Rpi-mcq1**	**Sw5**	

	Chromosome 10							
BIN	1-3	15-18	22-26	38-39	41-48	54	60-66	85-99
NBS1	0	0	0	0	1	1	3	0
NBS2	0	0	0	0	0	0	2	0
NBS5a6	1	0	0	0	1	0	1	0
NBS9	0	0	0	0	0	0	5	1
NBS13R	0	0	0	0	0	0	0	0
NBS15F	0	0	0	1	0	0	0	0
TIR300F	0	0	0	0	0	0	0	0
TIR300Fc	0	0	0	0	0	0	0	0
TIR3R	0	0	0	0	0	0	4	1
TIR9256	0	0	0	0	0	0	1	0
TIRWCF	0	0	0	0	0	0	0	0
chcF2	0	0	0	1	1	3	0	0
chcR1	0	0	0	6	4	1	0	0
chcR2	0	0	1	8	6	1	0	0
PK1Fa	0	0	1	0	0	0	1	0
PK1Fb	0	0	0	0	0	0	0	0
PK3Fb	0	3	0	0	0	0	1	0
PK4R1a	0	0	0	0	0	0	0	0
Brugmans & this study*	RH10.1			*RH10.2*	*RH10.3*	*RH10.4*		
Bakker**	10a						10bc	
Jupe***			C48	C47	C46	C45	C44	
R gene families****	Putative RGA			**Rpi-chc1**	Rpi-chc1	Rpi-chc1		

	Chromosome 11				Chromosome 12				
BIN	1-7	56-67	75-83	84-86	1-7	11-16	24-28	40-45	46-51
NBS1	0	2	9	19	0	1	0	0	8
NBS2	0	3	13	13	0	0	0	0	11
NBS5a6	0	6	1	15	0	0	1	0	3
NBS9	1	6	0	0	1	0	1	0	12
NBS13R	14	1	0	0	0	0	0	0	0
NBS15F	17	3	0	0	0	0	0	0	0
TIR300F	25	1	0	0	0	0	0	1	1
TIR300Fc	19	1	0	0	0	0	0	1	0
TIR3R	18	0	0	0	0	0	0	0	3
TIR9256	13	0	0	0	0	0	0	0	3
TIRWCF	19	0	0	0	0	0	0	4	3
chcF2	0	0	0	0	0	0	0	0	3
chcR1	0	0	0	0	0	0	0	0	0
chcR2	0	0	0	0	0	0	0	0	4
PK1Fa	0	0	0	0	0	0	0	0	1
PK1Fb	0	0	0	0	2	0	0	0	13
PK3Fb	0	0	0	0	0	0	0	6	7
PK4R1a	3	0	0	0	0	0	0	3	3
Brugmans & this study*	*RH11.1a*	RH11.1	RH11.2	RH11.3			*RH12.1*	*RH12.2*	*RH12.3*
Bakker**	11a	11bc	11d	11ef	12a				12b
Jupe***	C49	C52	C53-54	C55	C61-63				C56-60
R gene families****	**N, Bs4**	**R3a, R3b**	R3a, R3b	R3a, R3b			Rx, Gpa2	N, Bs4	**Rx, Gpa2**
