# Supplementary material for: Novel applications of motif-directed profiling to identify disease resistance genes in plants
Source: Plant Methods. 2013 Oct 7;9:37. doi: 10.1186/1746-4811-9-37 (PMC3853995; doi:10.1186/1746-4811-9-37)
Supplement: Additional file 2: Table S2 — Discrepancies between RGA clusters from RH and DM. [file 1746-4811-9-37-S2.pdf]

Clustername or position in RH	tags in RH	Cluster in DM	sequences in DM
RH1.3a			absent
chr2-3	too little sequence information for map alignment		
RH4 BIN70-79	few tags, none confirmed	C19	
4f*	few tags, none confirmed		absent
RH5 BIN4-12	many tags, none confirmed	C20-21	
RH5.3			singletons, no cluster assigned
RH7 BIN1-7	few tags, none confirmed	C34-35	
7b*	few tags, none confirmed		absent
RH8 BIN36-51	few tags, none confirmed	C39	
RH8 BIN73-81	few tags, none confirmed	C40-41	
9bc*			absent
9e*			absent
RH9.1a			singletons, no cluster assigned
RH9.1b	only CNL	C42	CNL + TNL
RH10.1			absent
10bc*	CNL and TNL	C44	only CNL
	no tags found	C48	
	no tags found	C50	
	no tags found	C51	
RH12.1			singletons, no cluster assigned
RH12.2	TNL		singletons, no TNL

* cluster names according to Bakker et al. 2011
