# Supplementary material for: Novel applications of motif-directed profiling to identify disease resistance genes in plants
Source: Plant Methods. 2013 Oct 7;9:37. doi: 10.1186/1746-4811-9-37 (PMC3853995; doi:10.1186/1746-4811-9-37)
Supplement: Additional file 3: Table S3 — Identification of unique and identical sequences within samples and among duplicate samples. [file 1746-4811-9-37-S3.pdf]

Sample	number of trimmed reads*	unique sequences within sample**	unique sequences among duplicates***
RH89-a	6280	2371	
RH89-b	3804	1620	572
sh83-a	3330	1443	
sh83-b	5147	2035	511
average	4640	1867	

* UID and NBS are trimmed at 5' side, successive 100nt are retained, 3' remaining sequence is discarded

** Sequences occurring at least once among trimmed reads

*** Sequences occurring at least once in both samples
