# Supplementary material for: Novel applications of motif-directed profiling to identify disease resistance genes in plants
Source: Plant Methods. 2013 Oct 7;9:37. doi: 10.1186/1746-4811-9-37 (PMC3853995; doi:10.1186/1746-4811-9-37)
Supplement: Additional file 4: Figure S1 — Example of comparable parts of two Li-Cor gels containing TIRWC-F primer RsaI and PK1Fb primer combined with MseI. Arrows indicate the positions of several polymorphic bands that were scored and mapped. Overall, the number of both polymorphic and monomorphic bands produced by PK profiling primers was lower as compared to TIR and NBS profiling markers. [file 1746-4811-9-37-S4.pptx]

## Slide 1
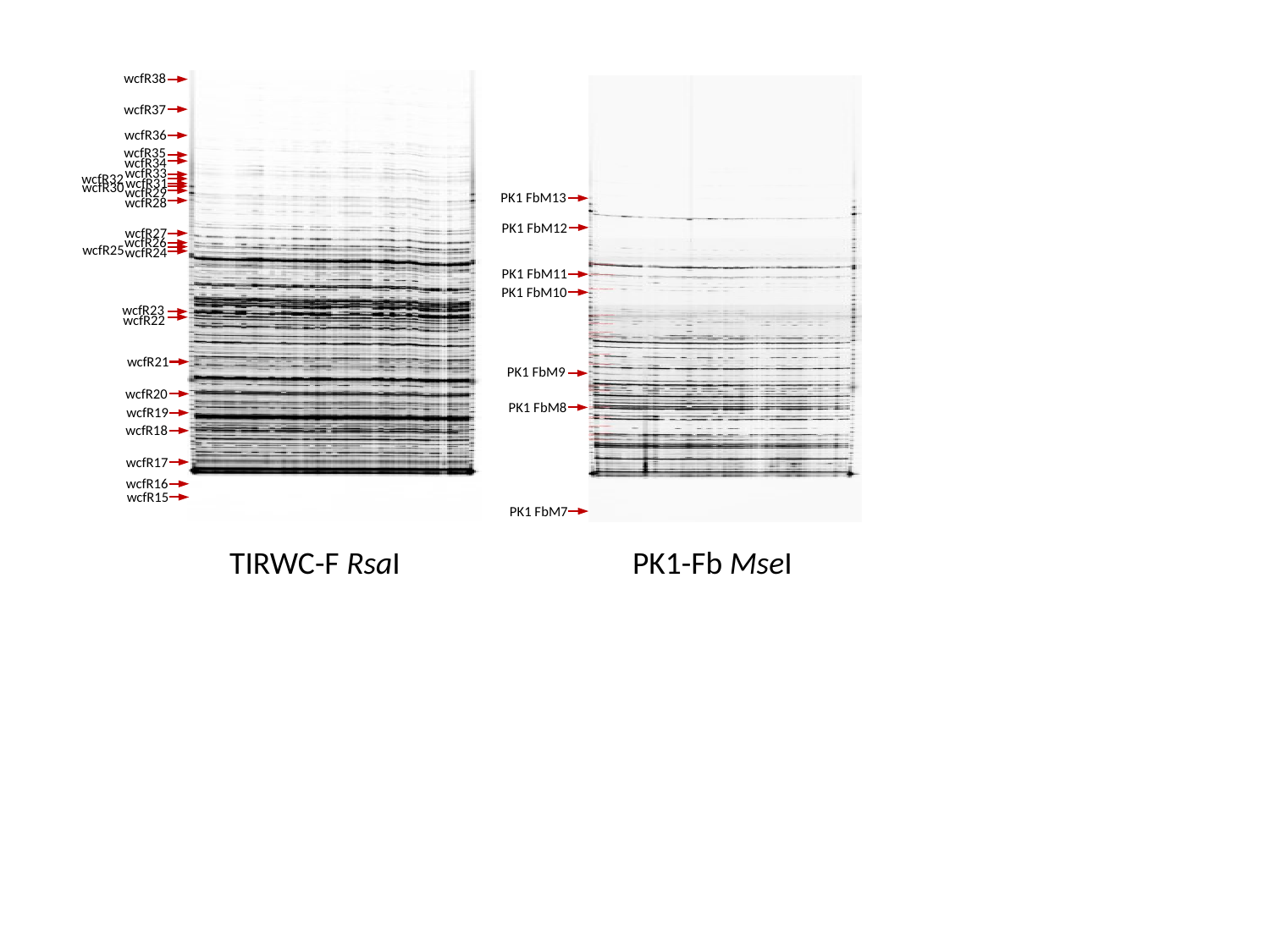

wcfR38
wcfR37
wcfR36
wcfR35
wcfR34
wcfR33
wcfR32
wcfR31
wcfR30
wcfR29
PK1 FbM13
wcfR28
PK1 FbM12
wcfR27
wcfR26
wcfR25
wcfR24
PK1 FbM11
PK1 FbM10
wcfR23
wcfR22
wcfR21
PK1 FbM9
wcfR20
PK1 FbM8
wcfR19
wcfR18
wcfR17
wcfR16
wcfR15
PK1 FbM7
TIRWC-F RsaI
PK1-Fb MseI
